# Triiodothyronine Increases mRNA and Protein Leptin Levels in Short Time in 3T3-L1 Adipocytes by PI3K Pathway Activation

**DOI:** 10.1371/journal.pone.0074856

**Published:** 2013-09-18

**Authors:** Miriane de Oliveira, Renata de Azevedo Melo Luvizotto, Regiane Marques Castro Olimpio, Maria Teresa De Sibio, Sandro José Conde, Carolina Biz Rodrigues Silva, Fernanda Cristina Fontes Moretto, Célia Regina Nogueira

**Affiliations:** 1 Department of Internal Medicine, Botucatu School of Medicine, University of São Paulo State (UNESP), Botucatu, São Paulo, Brazil; 2 Department of Physiology, Federal University of São Paulo (UNIFESP), São Paulo, Brazil; Virgen Macarena University Hospital, School of Medicine, Spain

## Abstract

The present study aimed to examine the effects of thyroid hormone (TH), more precisely triiodothyronine (T3), on the modulation of leptin mRNA expression and the involvement of the phosphatidyl inositol 3 kinase (PI3K) signaling pathway in adipocytes, 3T3-L1, cell culture. We examined the involvement of this pathway in mediating TH effects by treating 3T3-L1 adipocytes with physiological (P=10nM) or supraphysiological (SI=100 nM) T3 dose during one hour (short time), in the absence or the presence of PI3K inhibitor (LY294002). The absence of any treatment was considered the control group (C). RT-qPCR was used for mRNA expression analyzes. For data analyzes ANOVA complemented with Tukey’s test was used at 5% significance. T3 increased leptin mRNA expression in P (2.26 ± 0.36, p< 0.001), SI (1.99 ±0.22, p< 0.01) compared to C group (1± 0.18). This increase was completely abrogated by LY294002 in P (1.31±0.05, p< 0.001) and SI (1.33±0.31, p< 0.05). Western blotting confirmed these results at protein level, indicating the PI3K pathway dependency. To examine whether leptin is directly induced by T3, we used the translation inhibitor cycloheximide (CHX). In P, the presence of CHX maintained the levels mRNA leptin, but was completely abrogated in SI (1.14±0.09, p> 0.001). These results demonstrate that the activation of the PI3K signaling pathway has a role in TH-mediated direct and indirect leptin gene expression in 3T3-L1 adipocytes.

## Introduction

Thyroid hormones (THs) actions are especially important during development, this actions regulate the growth and maturation of many organs and tissues during the fetal and neonatal life [1,2]. Many tissues are regulated by THs up to their complete development, including actions on groups of genes involved in the differentiation program. The adipose tissue (AT) is an important target for THs. The AT is specialized in the transport, synthesis, storage, and mobilization of lipids. Its main function is the storage of energy in the form of triglycerides, and it constitutes a reservoir of energy to be used in times of caloric deprivation [3]. AT is the largest endocrine organ in the body, secreting hormones, chemokines, and cytokines (commonly referred to as *adipokines*) that are important paracrine/endocrine regulators [4].

The leptin is an AT product and together with THs are involved in the energy balance [5]. TH status also influences leptin, various studies try to correlate leptin with TH [6]. Administration of 3,5,3’- triiodothyronine (T3) to hypothyroid rats also decreased leptin mRNA expression in adipose tissue and circulating leptin levels [7]. However, in other reports, THs up-regulate leptin in differentiated adipocytes. T3, but not thyroxine (T4), stimulated ob mRNA expression and leptin secretion in 3T3-L1 adipocytes [8], cells in which leptin expression is less than 1% of intact adipose tissue. Additionally, human studies show no conclusive evidence on the relationship between TH and leptin levels [9,10].

The presence of thyroid hormone receptors (TRs) in AT suggests that there might be a direct interference of the thyroid activity in the production of adipokines at AT [11]. Studies in a microarray investigation, showed that the THs regulates 19 genes from humans white AT [12]. These genes originate proteins that are involved in signal transduction, lipid metabolism, apoptosis, and inflammatory responses.

Studies from our group showed that a T3 physiological dose increases leptin gene expression in obese animals subjected to diet restriction [13]. However, animals subjected to supraphysiological doses of T3 showed a diminishment in leptin mRNA expression [14]. These findings suggest that THs physiological levels might be necessary for the adequate leptin secretion [13]. Notwithstanding, our methodology did not enable us to verify if THs acts directly on AT modulating leptin expression.

The concept regarding the THs action has been diversified, including non-classical actions of T3 and T4. T3 has been shown as an activator of the phosphatidyl inositol 3-kinase (PI3K), which leads to an increase in certain genes, such as Hypoxia-inducible factor 1 (HIF-1α) and calcineurin inhibitor (ZAKI-4α) [15,16]. Hence, the present study assessed the effect of physiological and supraphysiological T3 doses in leptin mRNA and protein expression levels at 3T3-L1, adipocytes culture, after one hour of treatment.

We have evaluated the influence of protein synthesis in the leptin transcription rate regulated by T3 using the translation-inhibitor cycloheximide (CHX). LY294002 was used to check if the non-classical pathway (PI3K) was involved in the T3 action in the leptin mRNA levels. We have found that T3 increases the leptin expression levels in one hour (short time) of treatment, and such increase occurs directly in T3 physiological dose and indirectly in supraphysiological dose, but both depend on the activation of PI3K pathway.

## Materials and Methods

### 2.1: Chemicals and antibodies

Isobutylmethylxanthine (IBMX), dexamethasone, insulin, cycloheximide (CHX), triiodothyronine (T3), LY294002 (LY), Dimethyl Sulfoxide (DMSO), Sodium hydroxide (NaOH) and Charcoal Stripped fetal bovine serum (FBS) were purchased from Sigma (St Louis, MO, USA). Dulbecco’s modified Eagle’s medium (DMEM), fetal bovine serum and Antibiotic-Antimycotic 100X solution were purchased from Gibco BRL (Grand Island, NY, USA). The Rabbit polyclonal to leptin antibody (AB9749) and the mouse monoclonal GAPDH antibody (AB97069) were obtained from ABCAM (Abcam, Cambridge, UK). The anti-rabbit and anti-mouse secondary antibodies were purchased from Santa Cruz Biotechnology (CA, USA). Enhanced chemiluminescence (ECL) reagents were obtained Amersham Biosciences (NJ, USA).

### 2.2 Cell culture and differentiation

Mouse 3T3-L1 preadipocytes were obtained from the Cell Bank of Rio de Janeiro (Rio de Janeiro, RJ, Brazil), and were grown in polystyrene six-well plates, at 37°C in DMEM supplemented with 10% FBS (Sigma), and 1% Antibiotic-Antimycotic 100X solution (Gibco). After cell conﬂuence (designated as day 0), differentiation was initiated with 1µg/ml insulin, 1µM dexamethasone (DEX), and 0.5 mM IBMX in DMEM containing 10% FBS. After a 4-day incubation the culture media were replaced by DMEM supplemented with 10% FBS and 1 µg/ml insulin, and the cells were then fed every two days with DMEM containing 10% FBS. 3T3-L1 cells were fully differentiated by day 8. After differentiation, cells were incubated for 24 hour in DMEM supplemented with 10% Charcoal Stripped FBS (to deplete T3) and 1 µg/ml insulin. After incubation, cells were treated with: either physiological T3 dose (10 nM, named P group) or supraphysiological T3 dose (100 nM, named SI group) during one hour (1h). A non-treated group, only 0.1% NaOH (diluent T3), was used as control (C). For the one-hour time period, it was also formed groups of P and SI added with CHX (10µg/ml) [16] and groups that were added with LY (50µM) [16]. The inhibitors LY294002 and CHX were added to the medium 1 h before T3 treatment.

### 2.3: Oil red O staining

3 T3-L1 cells were grown on 6-well plates and induced to differentiate as previously described. After an 8-day incubation (day 8), plates were twice washed with phosphate-buffered saline (PBS), ﬁxed with 37% formaldehyde during 30 minutes at room temperature, and then washed twice again with PBS. After ﬁxation, cells were stained for 2h at room temperature with a ﬁltered of oil red O solution (0.5 g oil red O (Sigma) in 100 ml isopropanol), washed twice with distilled water and visualized to confirm differentiation.

### 2.4: Gene expression

Total RNA was extracted from 3T3-L1 cells by Trizol (Invitrogen) method, according to the manufacturer’s instructions. The High Capacity cDNA reverse transcription kit for RT-PCR® (Invitrogen, São Paulo, Brazil) was used for the synthesis of 20 µL complementary DNA (cDNA) from 1000 ng of whole RNA.

Leptin (assay Mm00434759_m1*- Applied Biosystems) levels were determined by RT-qPCR. Quantitative measurements were made in the “Applied Biosystems StepOne Plus” detection system using the TaqMan qPCR commercial kit (Invitrogen) according to the manufacturer’s instructions. Cycling conditions were as follows: enzyme activation at 50°C during 2 min, denaturation at 95°C during 10 min, cDNA products were ampliﬁed during 40 cycles of denaturation at 95°C during 15 s and annealing/extension at 60°C during 1 min. Gene expression was quantified in relation to the values of the C group after normalization by an internal control, cyclophilin (Mm00434759_m1), by the 2 ^− ΔΔCt^ method, as previously described [17].

### 2.5: Western blot analysis

Following treatment with T3, cells were washed twice with cold PBS, and lysed with 100 µl of lysis buffer containing 500 mM Tris (pH 8), 150 mM NaCl, 1% Triton X-100, 0.1% sodium dodecyl sulfate (SDS), and 0.5% deoxycholate of sodium. The homogenate was centrifuged at 4°C for 20 minutes at 12000 rpm. The supernatant was collected and total protein content was determined by the Bradford Method [18] using Albumin Serum Bovine (BSA) as standard. Samples containing 50 µg of protein were subjected to 15% SDS-polyacrylamide gel electrophoresis (SDS-PAGE) in polyacrylamide gels. After electrophoresis, proteins were electro-transferred to nitrocellulose membrane using a semidry transfer system (BioRad Biosciences; NJ, USC). The blotted membrane was then blocked 5% BSA in Tris buffered saline (TBS) with 0.1% Tween-20 (T) for 1 h at room temperature (RT) and incubated with specific antibodies overnight at 4°C. The primary antibodies used were rabbit polyclonal anti-leptin 1: 5000 and mouse monoclonal anti-GAPDH 1: 1000. After incubation with secondary anti-rabbit (1: 1000) and anti-mouse (1: 10000) in 5% BSA in TBS-T for 1h RT, blots were developed using the SuperSignal West Pico Chemiluminescent Substrate kit (Pierce) and exposed to Hyperﬁlm ECL (Amersham Bioscieneces). Quantitative analysis of immunoblot images was performed using Gel Logic (6000 PRO) with Carestream MI software. Results are shown as the squared pixels ratio between leptin and GAPDH, and represented as fold activation relatively to cells exposed to assay media.

### 2.6: Statistical analysis

Gene expression was analyzed using the analysis of variance (ANOVA) followed by Tukey’s test. Data are expressed as mean ± standard deviation. The significance level was set at 5%.

## Results

### 3.1: 3T3-L1 cell culture and differentiation

**Figure 1:**
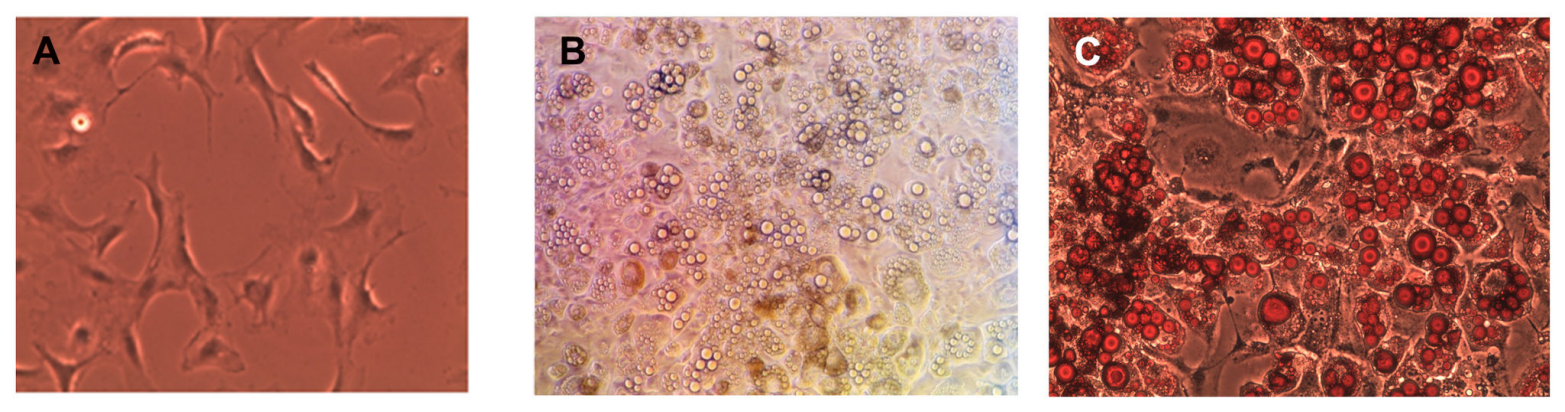
3T3-L1 cells before and after their differentiation into adipocytes. A) Non-differentiated cells. B) Cells with ten days of differentiation. C) Cells in *oil* *red* *O* after ten days differentiation. Arrows show adipocyte with cytoplasmic lipid droplet.

Figure 1A presents 3T3-L1 cells before differentiation. In the presence of a differentiation mix (insulin, dexamethasone, and IBMX) the pre-adipocytes turn into adipocytes presenting the morphology of mature adipocytes (Figure 1B and 1C), whose main characteristic is the presence of a great amount of cytoplasmic lipid droplets. *Oil red O* makes these droplets more evident and lipids are stained red (1C).

### 3.2: Leptin mRNA and protein levels increase after one hour of T3 incubation

**Figure 2:**
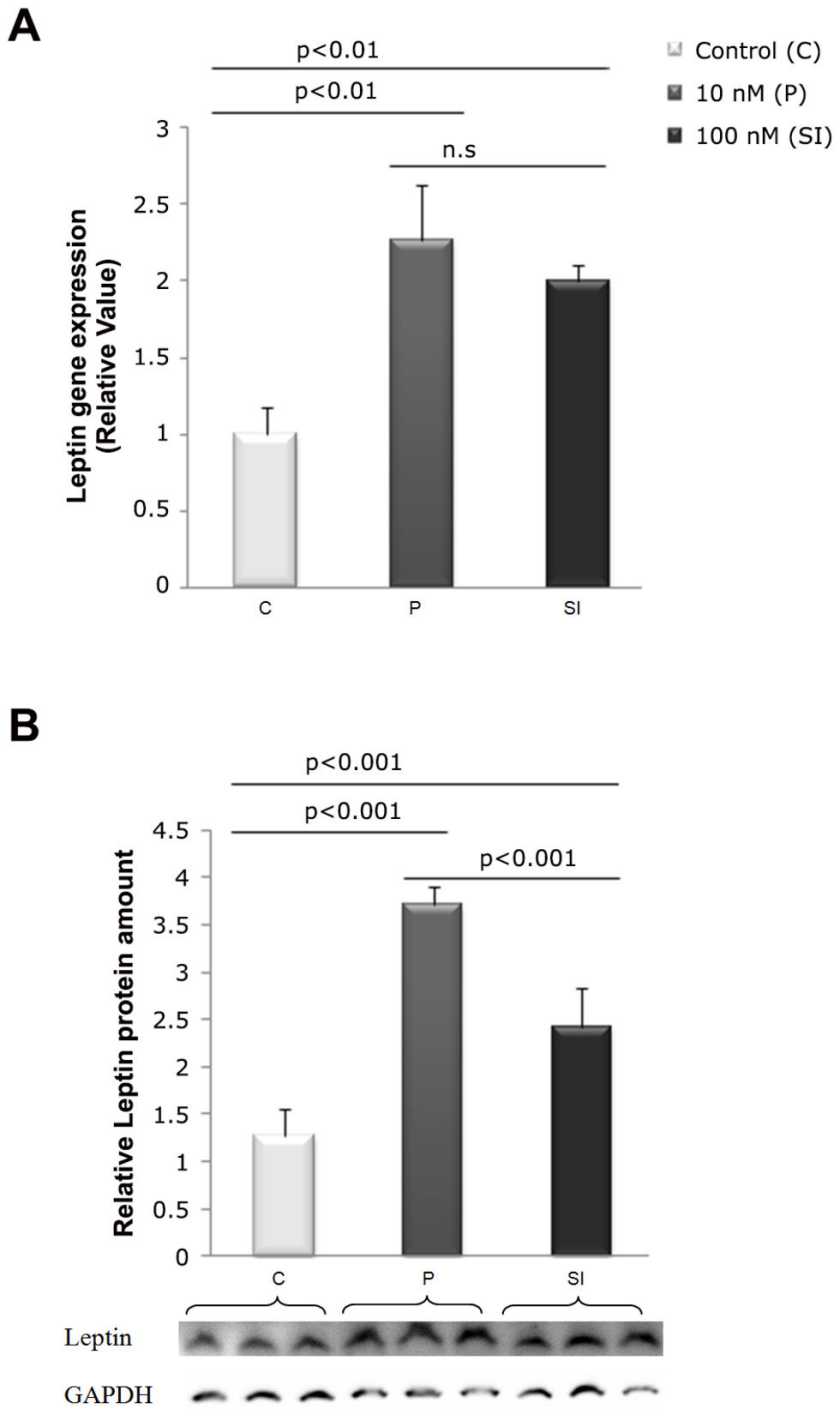
Dose-response for T3 effects upon leptin mRNA expression and protein in 1h. P =10 nM T3, SI = 100 nM T3, C = without T3. A) T3 effects in leptin mRNA expression by 10 nM and 100 nM doses. B) T3 effects in leptin protein by 10 nM and 100 nM doses. Data expressed in average and standard deviation (ANOVA supplemented with Tukey’s test). n.s = non-significant; n = 3 in each treatment.

Figure 2 shows that T3 increased the levels of leptin mRNA (Figure 2A) and protein (Figure 2B) in 3T3-L1 adipocytes in P and SI if compared to the C group after one hour of incubation.

### 3.3: The increase of Leptin mRNA and protein induced by T3 action is sensitive to LY294002

**Figure 3:**
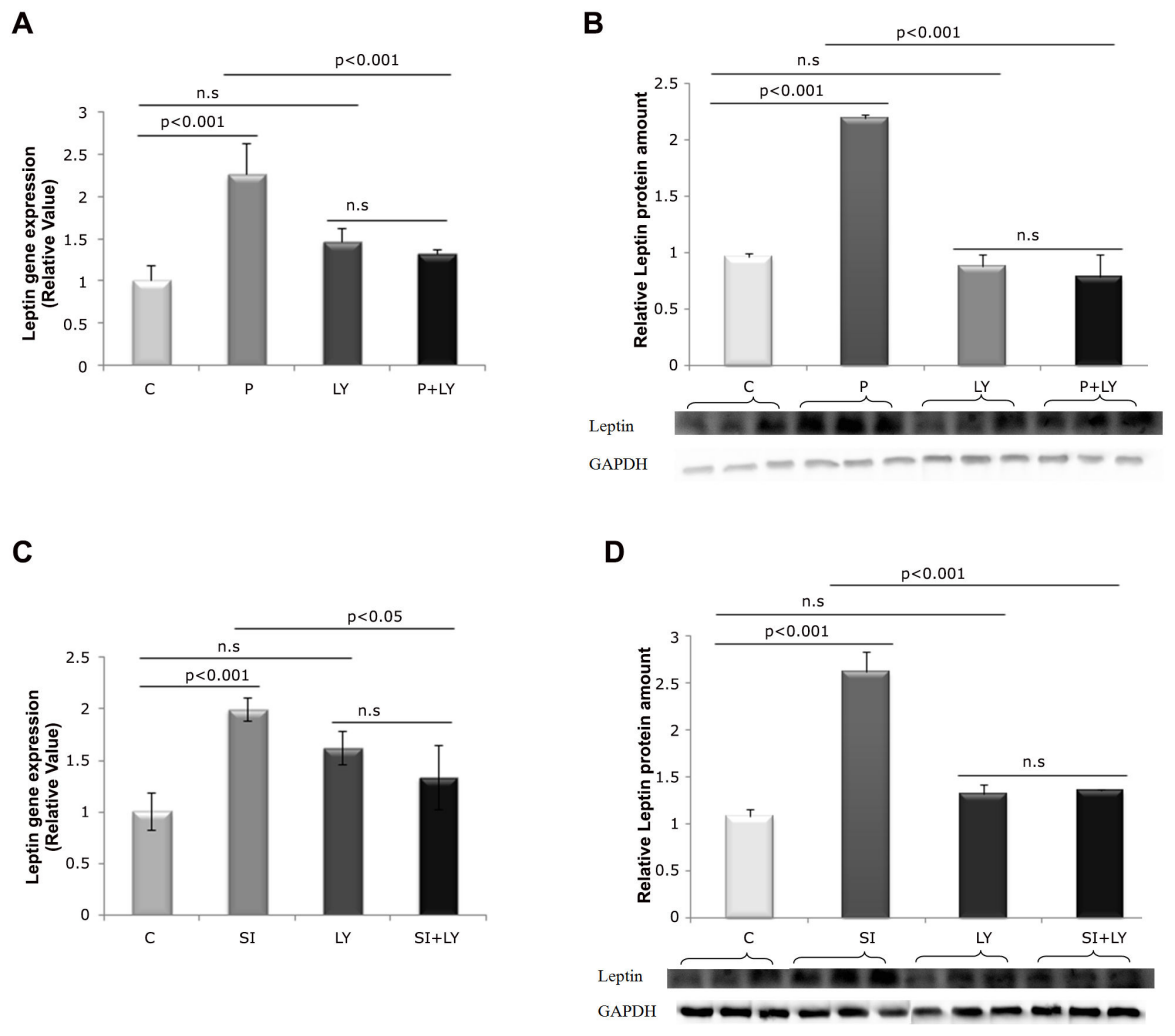
Effects of T3 and LY294002 upon the modulation of leptin mRNA and protein levels in 1h of treatment. P =10 nM T3, SI = 100 nM T3, C = without T3, LY = 50µM LY294002. A) T3 influence (10 nM) in the leptin gene expression under the presence/absence of LY. B) T3 influence (10 nM) in the leptin protein under the presence/absence of LY. C) T3 influence (100 nM) in the leptin gene expression under the presence/absence of LY. D) T3 influence (100 nM) in the leptin protein under the presence/absence of LY (ANOVA supplemented with Tukey’s test). n.s = non-significant; n = 3 in each treatment.

In order to check if PI3K pathway is involved in the mediation of leptin mRNA and protein expression after one hour of incubation with T3, groups P and SI were treated with a PI3K inhibitor (LY294002). Figure 3 shows that the addition of LY to different T3 doses led to the diminishment of leptin expression that were increased in P and SI (Figure 3A, B, C and D).

### 3.4: Effects of the protein synthesis inhibition upon the modulation of T3 induced leptin mRNA levels

**Figure 4:**
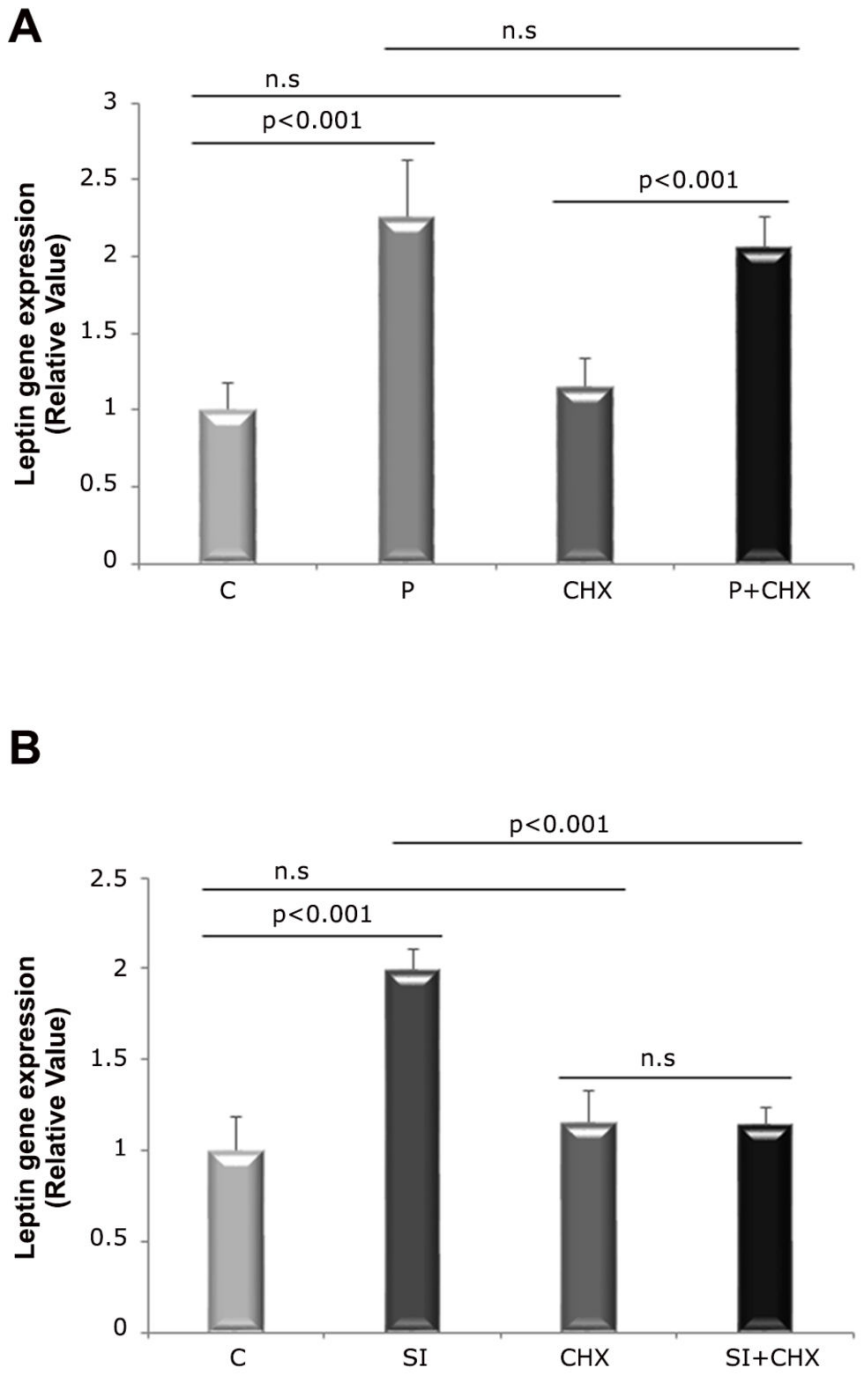
Effects of T3 and CHX upon the modulation of leptin mRNA levels in 1h treatment. P =10 nM T3, SI = 100 nM T3, C = without T3, CHX = 10µg/ml CHX. A) T3 influence (10 nM) in the leptin gene expression under the presence/absence of CHX. B) T3 influence (100 nM) in the leptin gene expression under the presence/absence of CHX (ANOVA supplemented with Tukey’s test). n. s = non-significant; n = 3 in each treatment.

In order to determine the need of protein synthesis for T3 regulation of leptin mRNA levels after one hour of treatment, groups P and SI were incubated with CHX. The addition of CHX to the P group did not change the levels of leptin mRNA (Figure 4A). However, CHX decreased the mRNA leptin levels in SI (Figure 4B).

##  Discussion

Since the adipose tissue is a target of thyroid hormones [19,20], some authors suggest that the leptin and thyroid might have a parallel response [21]. However, many studies investigating the possible relationship between thyroid hormone and leptin levels, reported conflicting results. Whereas some studies show no association between leptin serum concentration and thyroid hormone levels [6,22,23], others report a negative [24,25] or a positive correlation [26,27]. These divergent results may occur due to the use of different models, which demonstrate that THs might have different actions according to the model and the systemic factors involved. Thus, the present study aims to elucidate the AT response to different T3 doses on leptin mRNA expression without the interference of systemic factors.

Experimental model of 3T3-L1 cells (embryonic *Mus musculus* cells) (Figure 1A) were differentiated *in vitro* into adipocytes (Figure 1B and C). These cells represent a well-established adipogenesis model [28]. Our findings suggest that T3 has rapid effects in the modulation of leptin expression, since P and SI showed an increase in leptin gene expression (Figure 2A) and protein (Figure 2B) after 1 hour of T3 incubation. These results agree with the findings of Yoshida et al. [8], who were the first to measure the leptin secretion levels in 3T3-L1 cells in the presence of T3. However, they have observed an increase in leptin mRNA levels after 24 hours of incubation. Expression of leptin increases during adipocyte differentiation, and peaks as mature adipocytes fill with lipids [29] and in Jiang et al. [30] showed that administration of T3 to the diﬀerentiating 3T3-L1 cells enhanced the accumulation of triglyceride.

At physiological concentration of 10nM of T3, administered to the P group, there was na increased in both mRNA expression and secretion of leptin. Findings of Luvizotto et al. [13] suggest that physiologic levels of thyroid hormone are necessary for appropriate leptin expression and this dose of T3 could be used safely in the treatment of obesity because, at this level, T3 increases the expression of leptin, a hormone with central actions that cause a reduced food intake and an increase in energy expenditure, but does not decrease body protein.

Several studies have shown that neither chronic hyperthyroidism nor acute T3 treatment has affected serum leptin levels [31,32]. In the study of Luvizotto et al. [14,33], the supraphysiological dose of 50 fold greater than physiological levels, signiﬁcantly reduced leptin gene expression. In hyperthyroid patients, plasma leptin levels were unaffected by treatment for 12–28 weeks with methimazole [34]. These data indicate that, in humans, the plasma leptin status in individuals with relatively low plasma leptin levels is little affected by thyroid status. However, Yoshida et al. [8] and the present study reported that T3 enhanced the accumulation of leptin mRNA as well as leptin release by mouse 3T3-L1 cells, in supraphysiological concentration of 100nM of T3. One reason for the difference between *in vivo* and *in vitro* expressions is due the fact that leptin expression levels in 3T3-L1 and 3T3-F442A cells is less than 1% compared to intact adipose tissue [35,36].

It is known that THs may act by mechanisms other than the classical TRs/Thyroid Hormone Responsive Elements (TREs) [15]. These mechanisms can be called non-classical or non-genomic because their initiation sites may be in the plasma membrane, like the activation of integrin αvβ3 pathway, or in the cytoplasm, where the TH activates the mitogen-activated protein kinase (MAPK) or PI3K pathway. The initiation sites are proteins that are characterized as iodothyronine receptors [16,37].

PI3K participates in a wide variety of cellular process, including intracellular trafficking, organization of the cytoskeleton, cell growth and transformation, and prevention of apoptosis [38,39]. PI3K has a role in differentiation of several cell lines [40,41], including adipocytes.

We used specific inhibitor of PI3K, LY294002, signaling cascades regarding leptin expression, because T3 have been reported to activation at these signaling pathways to modulate specifics genes [15,16]. It was observed that leptin mRNA and protein levels that were T3-increased in P and SI, were then suppressed by the presence of LY294002 (Figure 3A, B, C and D), without cell toxicity. These findings strongly suggest that up-regulation of leptin gene transcription is mediated via the PI3K pathway by T3.

In order to check if the T3 action was direct or indirect upon the modulation of leptin gene, it was used a protein synthesis inhibitor in parallel with the use of a PI3K pathway inhibitor. The addition of CHX, which is a protein synthesis inhibitor, induced a leptin mRNA levels diminishment in group SI (Figure 4B), indicating that the effects of T3 upon this gene need protein synthesis prior to transcription. Under physiological T3 dose (group P) the CHX did not display any effect in leptin mRNA (Figure 4A), indicating that T3 has direct action upon the leptin mRNA expression increase. To our knowledge, this is the first study using CHX to determine the direct/indirect action of T3 upon the levels of leptin.

In summary, these data suggest that physiological and supraphysiological dose of T3 increase leptin mRNA expression after one hour of incubation without the characterization of a dose-dependent event. After one hour of T3 treatment, there is a direct increase in leptin levels when using physiological dose, whereas supraphysiological dose show an indirect leptin levels increase that depends on the PI3K pathway activation. Since leptin is an important adipostatic signal to the brain and it was observed that it is modulated by T3 through the activation of PI3K cytosolic pathway, the understanding of its mechanism of action is of utter importance.
